# Beneficial Effects of Pulsed Electromagnetic Field during Cast Immobilization in Patients with Distal Radius Fracture

**DOI:** 10.1155/2020/6849352

**Published:** 2020-02-25

**Authors:** Lucyna Krzyżańska, Anna Straburzyńska-Lupa, Patrycja Rąglewska, Leszek Romanowski

**Affiliations:** ^1^Physiotherapy Laboratory, J. Struś Municipal Hospital, Poznań, Poland; ^2^Department of Physical Therapy and Sports Recovery, Poznań University of Physical Education, Poznań, Poland; ^3^Traumatology, Orthopedics and Hand Surgery Department, Poznań University of Medical Sciences, Poznań, Poland

## Abstract

To assess whether pulsed electromagnetic field therapy during cast immobilization of distal radius fractures has beneficial effects on pain and limb function, the study included 52 patients (mean age 60.8 ± 15.0 years) with distal radius fractures treated with cast immobilization. Patients were allocated to a pulsed electromagnetic field group (*n* = 27) or a control group (*n* = 25). Pain; forearm and arm circumference; range of motion; disabilities of the arm, shoulder, and hand score; and touch sensation were evaluated on the day of the plaster cast dressing and 3 and 6 weeks after. In comparison to the control group, the pulsed electromagnetic field group reported significant changes after 3 and 6 weeks of treatment: lower pain levels (*p*=0.0052; *p* < 0.0001, respectively), greater mobility of upper-limb joints, improvement in exteroceptive sensation, and reduction in disability of the upper limb (disabilities of the arm, shoulder, and hand) (*p*=0.0003; *p* < 0.0001, respectively). Our results suggest that early addition of pulsed electromagnetic field treatment, during cast immobilization of distal radius fractures, has beneficial effects on the pain, exteroceptive sensation, range of motion, and daily functioning of patients.

## 1. Introduction

A distal radius fracture (DRF), also known as Colles' fracture, is one of the most common fractures [1] in people aged over 40 years [2].

The therapeutic procedure for DRFs consists of the repositioning of the fracture and cast immobilization. In some cases, surgery is performed. An important element of treatment is the rehabilitation intervention aimed at restoring the strength and function of the limb and helping prevent complications. However, a meta-analysis indicated that insufficient evidence is available to determine the best form of rehabilitation [1].

Early introduction of treatment in patients with DRFs aims to decrease pain and improve hand function. Collins [3] emphasized that DRF treatment needs to be implemented when the forearm is still immobilized to maximize functional recovery. During this period, joint mobilization exercises are often ignored, resulting in the reduction of range of joint motion (ROM), muscle atrophy, and pain [4]. Romanowski and Lisiewicz-Bręborowicz [5] reported the importance of paying attention to the joints, which are not covered with cast, in the early stage of physiotherapy. Metacarpophalangeal joints are particularly important because they are prone to contractures and pain. Performing finger joint motions and global motions is, therefore, advisable. Because of the cast weight, drawing the patient's attention to humeral joint motion, which is often neglected in the period of forearm cast immobilization, is important. However, it is a common practice to refer patients to rehabilitation only after cast removal [6–8]. The aim of physiotherapy is to strengthen the muscles, extend the joint ROM, and reduce edema [2].

Pulsed electromagnetic fields (PEMF) have been used for many years to support fracture healing [9]. A meta-analysis suggested that stimulation of the electromagnetic field may have some benefits in the treatment of bone nonunion or delayed union. However, the mechanisms of action at cellular and molecular levels have not been fully explained [9, 10].

In addition, PEMF therapy has shown other beneficial effects, including reduction of acute and chronic pain associated with connective tissue (tendon, ligaments, bone, and cartilage) injury as well as edema and inflammation control and wound healing [11].

Various strategies for the use of PEMF can be found in the literature. The studies mainly involve patients with tibia fractures [12–14]. Most studies describe starting PEMF treatment only when a nonunion is diagnosed: immediately after diagnosis of delayed union, about 16 weeks after the fracture, or at a late stage of delayed union, more than 6 months after the fracture [13, 14]. Griffin et al. [13] concluded that introduction of electromagnetic field stimulation for the treatment of delayed union and nonunion of long-bone fractures to current practice requires further investigation. Few studies describe the administration of this treatment during the acute stage of the fracture [15]. A single study investigated the clinical effectiveness of 10 PEMF treatments applied from day 7 after a Colles' fracture in postmenopausal women [16]. However, in this study, the authors did not assess how the therapy affected functioning of these patients in everyday life.

Therefore, this study aimed to determine the effect of PEMF therapy, started a day after injury and cast immobilization, on pain, edema, limb range of motion, exteroceptive sensation, and daily functioning in patients with DRFs.

## 2. Methods

### 2.1. Participants

The study included 52 consecutive patients (45 women and 7 men; mean age 60.8 ± 15.0) who were admitted to the casualty department after a distal radius fracture based on the AO classification (Arbeitsgemeinschaft für Osteosynthesefragen) developed by Müller et al. [17], type A and B according to the OTA (Orthopaedic Trauma Association) [18]. On the admission day, they were treated with reduction and cast immobilization after clinical and radiographic examination.

The inclusion criteria for the study were as follows: distal end radius fracture type A or B according to the AO classification, cast immobilization treatment, no limitation of wrist and hand function before injury, no contraindication to PEMF, and patient's consent. The exclusion criteria were multiple-organ injury; reports of previous DRF; and concurrent diseases influencing DRF recovery, such as diabetes, hyperthyroidism, psychiatric illness, and inflammatory osteoarthritis.

### 2.2. Ethical Considerations

This study was approved by the Research Ethics Committee at Poznań University of Medical Sciences (protocol number: 957/11). All procedures performed in the study were in accordance with the principles of the 1964 Declaration of Helsinki. Each participant was informed about the study and signed the informed consent statement.

### 2.3. Procedures

The study participants were allocated to one of the two groups: the PEMF group, consisting of 27 patients, and the control non-PEMF group, consisting of 25 patients.

The PEMF therapy device was produced by ASTAR ABR (Bielsko-Biała, Poland). The patient's hand, wrist, and distal forearm were placed inside a concentric coil applicator 345 mm in diameter and 440 mm in height generating a magnetic field intensity of 6–10 mT and a frequency of 25–30 Hz. Each treatment lasted 30 min. The magnetic field applied was initially generated for 1 s with a break. The length of the break in the first treatment was 3 s and was reduced by 0.5 s in each consecutive application. As from the seventh treatment, the magnetic field applied was generated with a constant amplitude throughout the treatment. For 10 days, the patients received the treatment once a day at the same time in the morning, with a break over the weekend, and then three times a week. Altogether, the patients received 22 treatments over six weeks [19].

On the day following the injury and cast immobilization, both the PEMF and control groups started rehabilitation that consisted of active shoulder, elbow, and finger mobilization exercises three times a day (all joints without immobilization). All participants were given instructions for home exercises to be performed under the supervision of a physiotherapist.

All patients participating in the study underwent conservative treatment without the need for surgery and the need for extension of the period of plaster immobilization beyond 6 weeks.

### 2.4. Outcome Measures

#### 2.4.1. Pain

The visual Analogue Scale (VAS) was used to assess pain severity. The results were obtained by measuring the distance (0 to 100 mm) from the beginning of the scale to the position selected by the patient, where “0” is “no pain” and “100” is “the worst possible pain” [20].

#### 2.4.2. Limb Circumference

Shoulder and forearm circumferences of both injured and uninjured limbs were measured with a tape measure. To assess edema, the measurement was taken at the proximal end of the radius (above the cast) and mid-shaft of the humeral bone at the deltoid tuberosity. The mean of the measurements of both limbs formed the outcome.

#### 2.4.3. Range of Joint Motion

The range of joint motion was measured using a goniometric method according to the rules of the International Standard Orthopaedic Measurement using the Sagittal Frontal Transverse Rotation system to an accuracy of 1°[21]. The range of motion of all joints without immobilization was measured three times. Mean measurement of both limbs formed the outcome. In joints that were not immobilized, the range of joint motion stayed within acceptable limits. The range of motion of the wrist was measured after cast removal and assessed in comparison to the uninjured limb.

#### 2.4.4. Grip Strength

Grip strength was measured using a Jamar dynamometer (Poland), and the results are expressed in kilograms (kg). The participant was seated comfortably in a chair, the shoulder was adducted and neutrally rotated, and the elbow was flexed at 90°, with the forearm and wrist in neutral positions. The participant was instructed to grip the dynamometer handle as hard as possible for 5 s [22]. Global grip strength was measured after cast removal and assessed in comparison to the uninjured limb.

#### 2.4.5. Touch Sensation

Two-point discrimination sense perception was measured using a standardized Dellon's discriminator (two-point discrimination test) with a preset distance between individual filaments as measurement points. The shortest distance was estimated at which two-point discrimination occurred (proper distance of 2–5 mm on a digital pulp) [23]. At the beginning of the examination, the patient was informed about the procedure. The examination was carried out statically in a patient whose eyes were closed. This manner of examination is believed to enable the evaluation of innervation density of slowly adapting touch sense receptors. The pads of fingers II and V were tested with one or two filaments. In the test, the patient was asked if they discriminated one or two points. When the pressure was sufficient, the skin around the pressure spot turned delicately white. Smaller distances, in mm, between the filaments resulted in the patient's improved ability to discriminate [24].

The WEST hand monofilament test (Weinstein Enhanced Sensory Test) (Bioinstruments, Connecticut, USA) was performed to assess the skin's sensitivity to touch, with the use of five individual fibers of standardized diameter, rigidity, and weight and with pressures of 0.07 g, 0.2 g, 4 g, and 200 g. The patient was sitting with eyes closed. Thumb and fingers II and V were examined, starting with the heaviest monofilament. The examination consisted of touching the given spot three times. It was considered successful when the patient could feel the touch at least one time [25]. Data analysis was categorized 1–5, with 1 (smallest force) standing for normal touch sensation. Larger figures indicate higher impairment of skin sensation [26].

#### 2.4.6. Disabilities of the Upper Limb

The DASH (disabilities of the arm, shoulder, and hand) questionnaire was used to assess the disability of the upper limb. In this questionnaire, the upper limb is considered a functional unity. Partial dysfunction of a limb impaired general function of a limb as well as everyday life and social activity of the patient. The basic version of the thirty-point questionnaire was used in this examination [27, 28].

The measurements were evaluated on the day of immobilization, then after 3 weeks, and on the day of the cast removal. All evaluations were carried out by the same researcher.

### 2.5. Statistical Analysis

Descriptive data were presented as median values and standard deviations. The normality distribution was tested using the Shapiro–Wilk test. Most variables in both groups were not distributed normally. To assess differences between the two groups, the Mann–Whitney *U* test was used. To assess the differences in the examination dates, the ANOVA Friedman's test was employed with the post-hoc comparison. The calculations were performed using StatSoft STATISTICA 10.0 software. A *p* value < 0.05 was considered significant.

## 3. Results

Patient demographics (Table 1) were comparable between the two groups.

Before the therapy, no significant difference was observed between the groups with regard to age, pain (VAS), circumference of the forearm and shoulder, and the range of motion (shoulder flexion, extension, abduction, and elbow flexion) (Tables 1 and 2).

Three and six weeks after the zero examination, significant pain (*p* < 0.01) and circumference of the forearm (*p* < 0.01) were reduced, whereas the range of motion significantly increased (shoulder flexion (*p* < 0.01), extension (*p* < 0.01), abduction (*p* < 0.01), and elbow flexion (*p* < 0.01)) in both groups.

Pain reduction was significantly higher during examination in the PEMF group in comparison to that in the control group (three weeks *p*=0.0052, six weeks *p* < 0.0001). No significant difference was noted between the two groups with regard to the circumference of the forearm and shoulder. The range of motion was significantly higher in the PEMF group in comparison to that in the control group: shoulder flexion (three weeks, *p*=0.0280; six weeks, *p*=0.0034), extension (three weeks, *p*=0.0004; six weeks, *p*=0.0004), and abduction (three weeks, *p*=0.0015; six weeks, *p*=0.0002) (Table 2).

Wrist mobility and global grip strength were evaluated only after cast removal at week six. In comparison to the control group, the PEMF group had significantly higher mean values for palmar and dorsal wrist flexion in the fractured limb (*p*=0.003 and *p*=0.001, respectively) and higher grip strength in the fractured and unfractured hand (*p*=0.0344 and *p*=0.0012, respectively) (Table 3).

Prior to therapy, no significant difference was found between the two groups with regard to the exteroceptive sensation measured with the WEST hand monofilaments and Dellon's discriminator.

After three weeks, a significantly greater improvement in exteroceptive sensation measured with Dellon's discriminator was observed in the PEMF group in comparison to that in the control group (*p*=0.0098). After six weeks, the exteroceptive sensation measured with the monofilaments and Dellon's discriminator was significantly better in the PEMF group in comparison to that in the control group (*p*=0.0013; *p*=0.0018, respectively) (Table 4).

The upper-limb disability evaluation (DASH) showed significant differences between the two groups in the first examination (*p*=0.0499). A significantly greater decrease in limb disability was recorded in the PEMF group in comparison to that in the control group after three (*p* < 0.0001) and six weeks of therapy (*p* < 0.0001) (Table 5).

## 4. Discussion

In comparison to the control group, the PEMF group showed a significantly higher reduction in pain, increased mobility of the upper-limb joint, improvement in exteroceptive sensation, and reduction in the upper-limb disability, assessed by the DASH questionnaire, after 3 and 6 weeks of treatment. In this study, the duration of casting was six weeks, and prolonging this duration was not necessary in any patient.

The study originally aimed to investigate the effects of the PEMF treatment, applied immediately after the fracture diagnosis and casting, on pain and limb function. In most published studies, except one [16], PEMF therapy was performed only after a nonunion had been diagnosed, more than three months after injury, with the bone union formation being primarily examined [14].

In a prospective randomized controlled study of long-bone fractures, Shi et al. [14] demonstrated that patients who received PEMF treatment between 16 weeks and 6 months of delayed union had a significantly increased rate of union and an overall reduction in the healing period compared to that achieved in those who received PEMF treatment after 6 months or more of delayed union. Griffin et al. [13] confirmed that the results of the conducted studies indicate the potential beneficial effect of magnetic fields on delayed union or nonunion. Meanwhile, they suggest further research for final conclusions.

Similarly, Hannemann et al. [15] concluded that the results of randomized studies on acute fractures are not enough to state that the beneficial effect of PEMF on bone growth stimulation also influences the reduction of nonunion cases. However, their systematic review and meta-analysis suggested possible beneficial PEMF effects, taking radiological and clinical union into consideration. Significant radiological union was observed in cases of acute inoperable fractures of the upper limb.

It should be noted that a DRF can lead to pain, edema, movement restrictions in joints, and functional impairment. Exercise and other physical interventions (also PEMF therapy for this purpose) are used to prevent complications and speed up recovery [1].

Several studies reported that the PEMF therapy has beneficial effects on both acute and chronic pain [29, 30].

The results of the present study confirmed the pain relief effect of magnetotherapy. Significantly lower pain levels were reported in PEMF patients after three and six weeks of therapy, referring to the initial examination, in comparison to the patients who were not exposed to magnetic field therapy. Our research results are in agreement with the observations reported by Lazović et al. [16] Their randomized study included 60 female patients with distal end radius fractures, divided into two groups. One group received 10 applications of 30-minute PEMF treatments, and the other was a control group. Each patient was taught and given instructions for a home-exercise program consisting of active shoulder, elbow, and finger exercises. The pain assessment values achieved in PEMF patients were lower, but not statistically significant [16].

In their systematic review of randomized studies, Andrade et al. [11] confirmed that the PEMF treatment causes pain relief and functional improvement in patients with low back pain. Hence, they reported the PEMF treatment as a potential alternative to pharmacological pain treatment.

In our research, significant edema reduction was reported in both groups, which is believed to be of primary importance in obtaining hand function [2]. Contrary to other studies [16, 29], we did not confirm a significant improvement in the PEMF group compared to control group. Similar to our research, Lazović et al. [16] applied PEMF to DRF patients during cast immobilization. In the research group, significant edema reduction was noted in comparison to that in the control group. Cheing et al. [31] achieved interesting results in their study. After cast removal, patients were randomized into four research groups, each receiving a combination of treatments, including ice packs, alternating magnetic fields, and simulated magnetotherapy. Results showed that the most significant reduction in edema was achieved by a combination of ice and magnetotherapy.

In our research, the beneficial effect of alternating magnetic fields on the increase in shoulder and elbow range of motion was observed. This can be explained by greater pain reduction in PEMF patients. Pain leads to increased muscular tension. Moreover, severe pain may have a negative impact on patients' psyche and, consequently, discourage them from participating in rehabilitation. The beneficial effect of PEMF on pain and function in musculoskeletal disorders has been confirmed in other studies [32].

Taking into consideration the wrist joint range of motion, the mean values for palmar and dorsal wrist flexion after fraction were significantly higher in the PEMF group in comparison to that in the control group. Significant differences were also noted in grip strength, which was measured after the period of cast immobilization.

Cheing et al. [31] noted a greater extended range of wrist flexion in patients who received magnetotherapy (either with or without ice) after cast removal. Similarly, Lazović et al. [16] found a statistically significant increase in palmar and dorsal wrist flexion in comparison to that in the control group.

Evaluation of exteroceptive sensation is vital for the assessment of hand function. Melchior et al. [33] demonstrated the relationship between upper-limb function and exteroceptive sensation, measured with Semmes-Weinstein monofilaments. Accessible literature provides individual studies about the effect of magnetotherapy on sensory threshold changes. Witkoś et al. [34] demonstrated a significant increase in sensory sensitivity in patients who received a single application of magnetotherapy in comparison to that in the control group.

In our own research, a significant increase in exteroceptive sensation, evaluated with Dellon's discriminator and monofilaments, was observed after six weeks in both groups. However, it was more significant in the PEMF group than in the control group. Notably, the weakening in touch sensation may have been as a result of soft tissue injury and pain that accompanied the fraction.

The authors of other studies point out further consequences of the fracture: functional impairment and loss of independence [1]. Therefore, DRF is not only a common clinical problem, but also a social and an economical one [35]. It entails high costs concerning treatment, rehabilitation, or absence from work. Therefore, it has been investigated for years which rehabilitation intervention is necessary to optimise functional recovery to achieve the activities required for daily living [1]. We were also interested in this aspect of rehabilitation with PEMF use.

The DASH questionnaire was used for assessment of upper-limb disability. It is considered one of the best clinical measures of the upper-limb disability assessment [36]. Its usefulness has been mentioned by many researchers [37–39]. Our results showed a decreased disability in subjective assessments after early physiotherapy. At the same time, a significantly lower disability index was recorded in the PEMF group than that in the control group after three and six weeks of therapy. A similar observation of the beneficial effect of early physiotherapy on hand function (DASH) after a displaced DRF was noted by Wilcke and Abbaszadegan [40].

The limitation of this study was the small number of patients included. However, 52 patients were examined three times by the same researcher, and all of them completed the examination. Our research did not only focus on the monitoring of symptoms associated with the pathology, but, more importantly, it evaluated the effect of the therapy on patients' daily functioning. The effect of PEMF treatment on fracture healing has not been studied.

In conclusion, this finding suggests that the early addition of PEMF treatment during cast immobilization for DRFs has some beneficial effect, particularly on the pain and function of the broken limb, and improves daily functioning. Final conclusions, however, await further well-conducted randomized controlled trials, in line with the principles of evidence-based medicine in this field.

## Figures and Tables

**Table 1:** Baseline characteristics of the investigated groups.

	PEMF group *N* = 27	Control group *N* = 25	*p* between groups
Gender (female/male)	22/5	23/2	
Age (years)	58.1 (18.2)	63.6 (10.4)	0.5275
Broken limb (right/left)	9/18	12/13	

PEMF: pulsed electromagnetic fields.

**Table 2:** Differences in the evaluation of pain, circumference, and range of motion between the PEMF and control groups during the study.

		PEMF group (*n* = 27)	Control group (*n* = 25)	*p* between groups
Pain VAS (mm)	Before	6.2 ± 2.00	7.1 ± 1.88	0.0956
	After 3 weeks	2.1 ± 1.41	3.5 ± 1.71	0.0052
	*p* value I *vs.* II	*p* < 0.01	*p* < 0.01	
	After 6 weeks	0.2 ± 0.42	2.7 ± 2.15	<0.0001
	*p* value I *vs.* III	*p* < 0.01	*p* < 0.01	

Circumference of forearm (cm)	Before	25.8 ± 2.46	25.9 ± 1.89	0.9198
	After 3 weeks	24.7 ± 2.39	24.2 ± 1.94	0.5097
	*p* value I *vs.* II	*p* < 0.01	*p* < 0.01	
	After 6 weeks	23.9 ± 2.31	22.9 ± 1.95	0.1285
	*p* value I *vs*. III	*p* < 0.01	*p* < 0.01	

Circumference of shoulder (cm)	Before	28.1 ± 2.54	28.9 ± 3.51	0.4582
	After 3 weeks	26.9 ± 2.52	27.6 ± 3.34	0.5097
	*p* value I *vs.* II	*p* < 0.01	*p* < 0.01	
	After 6 weeks	26.4 ± 2.36	26.8 ± 3.41	0.7486
	*p* value I *vs.* III	*p* < 0.01	*p* < 0.01	

Range of motion (°) shoulder flexion	Before	151.8 ± 18.96	146.4 ± 17.32	0.1331
	After 3 weeks	162.1 ± 12.78	156.6 ± 13.87	0.0280
	*p* value I *vs.* II	*p* < 0.01	*p* < 0.01	
	After 6 weeks	167.5 ± 6.42	162.0 ± 9.43	0.0034
	*p* value I *vs.* III			

Shoulder extension	Before	39.6 ± 5.23	37.8 ± 6.03	0.1935
	After 3 weeks	47.7 ± 3.11	42.6 ± 5.19	0.0004
	*p* value I *vs.* II	*p* < 0.01	*p* < 0.01	
	After 6 weeks	49.7 ± 0.81	45.8 ± 4.29	0.0004
	*p* value I *vs.* III	*p* < 0.01	*p* < 0.01	

Shoulder abduction	Before	149.9 ± 18.21	142.3 ± 14.12	0.0727
	After 3 weeks	164.5 ± 8.19	153.6 ± 12.7	0.0015
	*p* value I *vs.* II	*p* < 0.01	*p* < 0.01	
	After 6 weeks	168.4 ± 3.72	159.5 ± 10.01	0.0002
	*p* value I *vs.* III	*p* < 0.01	*p* < 0.01	

Elbow flexion	Before	116.1 ± 14.10	111.2 ± 8.55	0.2198
	After 3 weeks	129.8 ± 14.10	122.8 ± 7.43	0.0115
	*p* value I *vs.* II	*p* < 0.01	*p* < 0.01	
	After 6 weeks	141.6 ± 7.19	132.1 ± 8.24	0.0001
	*p* value I *vs.* III	*p* < 0.01	*p* < 0.01	

PEMF: pulsed electromagnetic fields; VAS : Visual Analogue Scale.

**Table 3:** Differences in wrist mobility and global grip strength between the PEMF and control groups after therapy.

	PEMF group (*n* = 27)	Control group (*n* = 25)	*p* between groups
Range of wrist dorsal flexion (°)			
Broken limb	26 ± 10.60	15 ± 6.85	0.0001
Unaffected limb	50 ± 0.0	50 ± 0.0	0.9927

Range of palmar flexion (°)			
Broken limb	29.5 ± 9.17	19.8 ± 5.79	0.0003
Unaffected limb	60 ± 0.0	60 ± 0.0	0.9927

Global grip strength (kg)			
Broken limb	4.5 ± 3.14	2.2 ± 1.52	0.0012
Unaffected limb	25.4 ± 10.72	19.4 ± 7.04	0.0344

PEMF: pulsed electromagnetic fields.

**Table 4:** Differences in the evaluation of exteroceptive sensation between the PEMF and control groups during the study.

Exteroceptive sensation		PEMF group (*n* = 27)	Control group (*n* = 25)	*p* between groups
Monofilaments (g)	Before	2.1 ± 0.72	2.2 ± 0.41	0.7555
	After 3 weeks	1.7 ± 0.55	1.9 ± 0.49	0.1585
	*p* value I *vs.* II	n.s.	n.s.	
	After 6 weeks	1.0 ± 0.19	1.6 ± 0.51	0.0013
	*p* value I *vs.* III	*p* < 0.01	*p* < 0.01	

Dellon's discriminator (mm)	Before	2.4 ± 0.74	2.6 ± 0.65	0.3051
	After 3 weeks	1.7 ± 0.66	2.3 ± 0.75	0.0098
	*p* value I *vs.* II	*p* < 0.05	n.s.	
	After 6 weeks	1.2 ± 0.42	1.8 ± 0.52	0.0018
	*p* value I *vs.* III	*p* < 0.01	0.01	

PEMF: pulsed electromagnetic fields.

**Table 5:** Upper-limb disability evaluation (DASH) in both groups during the study.

Disabilities of the upper limb		PEMF group (*n* = 27)	Control group (*n* = 25)	*p* between groups
DASH questionnaire [score]	Before	67.6 ± 13.16	75 ± 17.06	0.0499
	After 3 weeks	45.9 ± 15.39	66.7 ± 19.39	0.0003
	*p* value I *vs.* II	<0.0001	<0.0001	
	After 6 weeks	29.1 ± 13.97	60.1 ± 20.29	<0.0001
	*p* value I *vs.* III	<0.0001	<0.0001	

PEMF: pulsed electromagnetic fields; DASH: disabilities of the arm, shoulder, and hand.

## Data Availability

The data used to support the findings of this study are available from the corresponding author upon request.
